# Developmental defects and behavioral changes in a diet-induced inflammation model of zebrafish

**DOI:** 10.3389/fimmu.2022.1018768

**Published:** 2022-10-26

**Authors:** Saima Rehman, Adnan H. Gora, Shubham Varshney, Jorge Dias, Pål A. Olsvik, Jorge M. O. Fernandes, Sylvia Brugman, Viswanath Kiron

**Affiliations:** ^1^ Faculty of Biosciences and Aquaculture, Nord University, Bodø, Norway; ^2^ SPAROS Lda, Olhão, Portugal; ^3^ Department of Animal Sciences, Host Microbe Interactomics, Wageningen University, Wageningen, Netherlands

**Keywords:** soybean meal, zebrafish, behavior, β-glucan, inflammation

## Abstract

Soybean meal evokes diet-induced intestinal inflammation in certain fishes. Although the molecular aspects of soybean-induced intestinal inflammation in zebrafish are known, the impact of the inflammatory diet on fish behavior remain largely underexplored. We fed zebrafish larvae with three diets - control, soybean meal and soybean meal with β-glucan to gain deeper insight into the behavioral changes associated with the soybean meal-induced inflammation model. We assessed the effect of the diets on the locomotor behavior, morphological development, oxygen consumption and larval transcriptome. Our study revealed that dietary soybean meal can reduce the locomotor activity, induce developmental defects and increase the oxygen demand in zebrafish larvae. Transcriptomic analysis pointed to the suppression of genes linked to visual perception, organ development, phototransduction pathway and activation of genes linked to the steroid biosynthesis pathway. On the contrary, β-glucan, an anti-inflammatory feed additive, counteracted the behavioral and phenotypic changes linked to dietary soybean. Although we did not identify any differentially expressed genes from the soybean meal alone fed group vs soybean meal + β-glucan-fed group comparison, the unique genes from the comparisons of the two groups with the control likely indicate reduction in inflammatory cytokine signaling, inhibition of proteolysis and induction of epigenetic modifications by the dietary glucan. Furthermore, we found that feeding an inflammatory diet at the larval stage can lead to long-lasting developmental defects. In conclusion, our study reveals the extra-intestinal manifestations associated with soybean meal-induced inflammation model.

## Introduction

Intestinal inflammation is a significant health problem that affects a considerable portion of the world population (1). A myriad of genetic and environmental factors have been associated with the onset of the disease (2) and intestinal inflammation can adversely affect the functions of other organs. For instance, chronic intestinal inflammation can lead to the development of several forms of psychiatric disorders (3–5). Mice models have been employed to understand the aftereffects of intestinal inflammation (6). For example, stress-associated behavior of chemically-induced colitis model (7, 8) and compromised cognitive ability after the consumption of high-fat inflammatory diet (9).

Similar to mice, zebrafish is a model organism widely used to understand intestinal inflammation (10, 11). This model replicates inflammation hallmarks like increased intestinal permeability, immune cell recruitment and alteration in the microbiota profile (12–14). An inflammation model of zebrafish has been developed by feeding zebrafish larvae at 5 days post-fertilization (dpf) with a soybean meal-based diet (12, 15). The success of the model could be due to triggering of an inflammatory reaction in the intestine of zebrafish larvae by saponins, which are antinutritional factors in soybean meal (12). This model is used extensively and mainly to understand the molecular aspects of diet-induced inflammation (15, 16). However, the extra-intestinal manifestations of this model have not been extensively studied. We believe that zebrafish is an ideal model to study diet-induced changes in swimming behavior.

Zebrafish larvae start to swim freely at around 4 dpf and their swimming behavior is modulated by both internal and external stimuli (17). At this early stage, larvae have a narrow repertoire of discrete stereotyped movements, which can be assessed to understand their behavior. With the use of automated movement tracking systems, it is now possible to monitor the activity of an individual and split a particular movement into several measurable parameters like distance travelled, movement, heading and turn angle which can be assessed to understand behavioral changes. In the present study, we analyzed the locomotor behavior of zebrafish larvae to understand the behavioral changes associated with a well-established soybean meal-induced inflammation model (12, 15). Additionally, we exploited transcriptomics data to gain deeper insight into the underlying molecular aspects of behavioral changes in the larval zebrafish model. Since it is well-known that β-glucan can impart anti-inflammatory effects (18, 19), we tested the efficacy of a commercial product to abate soybean meal-induced behavioral changes.

## Materials and methods

### Zebrafish husbandry

Adult zebrafish (AB strain) were maintained in a recirculatory aquaculture system at Nord University, Norway, following standard protocols (20). Zebrafish eggs were obtained by naturally breeding sexually mature males and females. Fish in five tanks were used for breeding, and in each of these tanks there were 15 males and 30 females. They were community bred and 300-400 eggs were obtained from each tank. These eggs were kept in larval rearing tanks (3.5 L) which were part of a freshwater flow-through system (Zebtec Toxicological Rack, Tecniplast, Varese, Italy), hereafter called system water. The eggs were randomly distributed into 18 rearing tanks, with 100 eggs in each tank. The water temperature in the tanks was 28 ± 0.5°C, the water flow rate was 1 L/h, and dissolved oxygen was 7-8 ppm (oxygen saturation > 85%). A 14L:10D photoperiod was maintained throughout the experimental period.

### Test diets and feeding study

Five-day-old larvae (five days post fertilization, 5 dpf) were used for the study and the test diets were prepared by SPAROS Lda (Olhão, Portugal). The control diet, CT, was a fish meal-based diet with high-quality marine protein. Soybean-based diet (SBM) contained 50% of the test component to induce a pro-inflammatory effect (12, 15). The β-glucan diet (BG) was supplemented with 2.5% (w/w) of the product Aleta^™^ (derived from the microalga *Euglena gracilis*; Kemin, Des Moines, USA) in the SBM diet (**Supplementary Table 1**). Each experimental fish group (6 tanks/group) was assigned to the respective test diet (< 100 µm particle size) from 5 to 14 dpf. From 15 dpf to 30 dpf, the larvae in the SBM, BG and CT groups were offered the control diet (100-200 µm particle size) (**Supplementary Figure 1**). The larvae were hand-fed four times a day *ad libitum*, i.e., at 08:00, 12:00, 16:00 and 20:00. The feeding study that started with 5-day-old larvae ended at 30 dpf.

### Sampling

At 15 and 30 dpf, larvae were analyzed under the microscope. In addition, their behavior was assessed, and oxygen consumption was recorded. Samples were collected at 15 dpf for transcriptomics. The larvae were immersed in a lethal dose of 200 mg/L of tricaine methanesulfonate (Argent Chemical Laboratories, Redmond, WA, USA) buffered with an equal amount of sodium bicarbonate. Five larvae were pooled to obtain one sample and 6 replicate samples were prepared from each treatment group. The samples were snap-frozen in liquid nitrogen and then stored at -80°C until further analyses.

### Microscopic examination

Larvae from each group (*n*=9-10) were randomly selected for the microscopic examination. For the study, the larvae were immobilized on a cavity glass slide using 3.5% (w/v) methylcellulose (Sigma Aldrich, Saint Louis, USA). Images were captured using a stereomicroscope (SZX12, Olympus, Shinjuku, Japan) equipped with an Olympus SC50 camera (Olympus Soft Imaging Solutions, Münster, Germany). Key morphological traits like standard length, snout-vent length, head-trunk angle, swim bladder area and eye area (21, 22) were measured using the *ImageJ* software (23).

### Locomotor behavior test

Larval locomotor behavior was assessed using the DanioVision system (Noldus Information Technology, Wageningen, the Netherlands). The assessment was performed as described in our previous study (24). The larvae were first acclimatized to 24 well plates for one hour at 28°C in an incubator (Sanyo MIR-154, Osaka, Japan). The analysis was carried out three times in the DanioVision system (*n*=20). The temperature of the well plates (28 ± 1°C) was maintained using the DanioVision temperature control unit. The 20 min behavior analysis included a 5 min dark period followed by a 5 min light period and then a second cycle of 5 min of darkness followed by 5 min of light period. The video recordings were analyzed using the EthoVision^®^ XT 16.0 software (Noldus Information Technology) to assess the distance moved, velocity, movement, angular velocity and heading angle.

### Sudan black staining

Zebrafish larvae (*n*=24-25; 15 dpf) were fixed overnight in 4% formaldehyde in PBS at 4 °C. After the fixation step, larvae were washed with cold PBS containing 0.1% Tween 20 (PBT) (Sigma Aldrich, Saint Louis, USA), and incubated in Sudan Black stain (Sigma Aldrich) for 20 min. Then the samples were washed (3-4 times; each time 10 min) in 70% ethanol. Larvae were then rehydrated with PBT and mounted in 90% glycerol for viewing under a stereomicroscope (SZX12, Olympus). Thereafter, the images were captured using Olympus SC50 camera (Olympus Soft Imaging Solutions) and analyzed to quantify the granulocytes in the mid and posterior intestine.

### Oxygen consumption analysis

Oxygen consumption of the larvae was determined using the Loligo^®^ Microplate Respirometry System (Loligo Systems, Viborg, Denmark). Twenty-four hours before the measurement, the instrument was calibrated with oxygen-saturated and oxygen-depleted system water at 28°C. The oxygen-depleted water was first prepared by dissolving 20 g of sodium sulfite (Sigma Aldrich) in 1 liter of system water. Then larvae from each treatment (*n*=12) were placed in the two independent 24-well plate sensor dishes (PreSens, Regensburg, Germany), with each well containing one larva. Thereafter, the 24-well plate sensor dish was submerged in a tank containing system water. During the 2 hour-long respiration measurement, the tank and plates were kept at 28°C in a climate chamber. The oxygen saturation was recorded using the software MicroResp^®^ version 1.0.4 (Loligo).

### RNA isolation, library preparation and mRNA sequencing

Total RNA was extracted from the frozen samples using the Direct-zol™ RNA MiniPrep kit (Zymoresearch, CA, USA) following the manufacturer’s instructions. The RNA concentration and integrity were determined using the Invitrogen Qubit 3.0 fluorometer (ThermoFisher Scientific, USA) and Tape Station 2200 (Agilent Technologies, Santa Clara, CA, USA). RNA from the samples (*n*=6) with RIN value ≥7 were used for RNA-Seq. Library preparation and sequencing were done by Novogene Europe, Cambridge, United Kingdom. Messenger RNA was purified from total RNA using poly-T oligo-attached magnetic beads. After fragmentation, the first strand cDNA was synthesized using random hexamer primers followed by the second strand cDNA synthesis. The libraries were end repaired, A-tailed, adapter ligated, size selected, amplified, and finally purified. The libraries were quantified using Qubit and real-time PCR and bioanalyzer was employed to detect the size distribution. The barcoded libraries were then pooled and loaded on the Illumina NovaSeq 6000 Sequencing system (Illumina, San Diego, CA, USA) to obtain 150 bp paired end reads. For each sample, a minimum of 20 million paired raw reads were obtained with an average of 21.4 million reads per sample. Overall, the average mapping percentage of filtered reads was 88% (**Supplementary Table 2**).

### Transcriptome data analyses

The quality of raw reads was assessed using the *FastQC* command line, and reads were filtered based on the Phred quality score (Q ≥ 30) using the tool *fastp* (25) (**Supplementary Table 2**). The filtered reads were then aligned to the reference zebrafish genome downloaded from NCBI (release 106) using *HISAT2*, version 2.2.1 with default parameters (26). *featureCounts* version 1.5.3 was employed to obtain the read counts that belong to each gene (27). Differential expression of the genes across the treatment groups was determined by *DESeq2* (28). Transcripts with |Log_2_ fold change| ≥ 1 and an adjusted *p*-value < 0.05 (Benjamini-Hochberg multiple test correction method) were considered significantly differentially expressed. The gene ontology (GO) and Kyoto Encyclopedia of Genes and Genomes (KEGG) pathway enrichment analyses were performed with the software *DAVID* (Database for Annotation, Visualization and Integrated Discovery) version 6.8 with *p* value of 0.05 and minimum gene count of 2 (27). GO term-gene networks were generated using *Cytoscape* version 3.8.2 (29). The packages *ggplot2*, *pheatmap*, *GOplot*, in R were employed to visualize the parameters of interest.

### Statistical analysis

The behavioral, morphological and granulocyte data were checked for the assumptions of normality (Shapiro-Wilk) and homogeneity of variance (Bartlett’s test). Parametric *t*-test and one-way ANOVA were performed where the normality assumptions were met. In the case of non-parametric data, statistical differences were identified using the Wilcoxon-Mann-Whitney test and Kruskal-Wallis test. Tukey’s test (parametric data) and Dunn’s test (non-parametric data) were employed to understand the statistical differences between treatments. We employed the gam function in the *mgcv* package of R to study the oxygen depletion in the three groups. In addition, we employed the *gganimate* package to create a gif to display the differences at 15 and 30 dpf separately. The angular data was analyzed using circular package in R and the statistical differences were detected using Watson U^2^ test.

## Results

### Morphological changes, locomotor activity, granulocyte number and oxygen consumption

**Figure 1:**
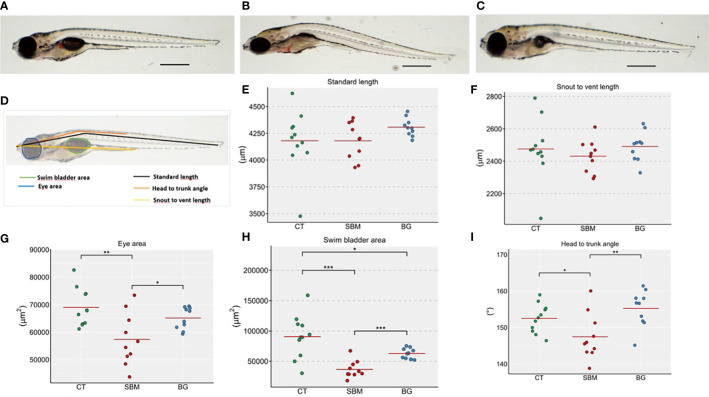
Diet-induced morphological changes in zebrafish larvae. Representative images of the zebrafish larvae fed the **(A)** CT **(B)** SBM and **(C)** BG diets **(D)**. Measurement strategy that was adopted to assess the morphological changes in zebrafish larvae. The measured parameters include **(E)** Standard length **(F)** Snout to vent length **(G)** Eye area **(H)** Swim bladder area **(I)** Head to trunk angle. Asterisks *** indicate p < 0.001, ** indicate *p* < 0.01, * indicates *p* < 0.05. Larvae were assessed at 15 dpf (n = 9-10 per group). Control - CT, soybean - SBM and β-glucan - BG. Scale bar = 500 μm.

Microscopic evaluation helped us to understand the diet-induced changes in the morphology of zebrafish larvae (**Figures 1A-D** and **Supplementary Figure 2**). The standard length and snout-vent length of the treatment groups did not differ significantly (**Figures 1E, F** **)**. However, we found a significant decrease in the eye area (**Figure 1G**) of the SBM group (*p* < 0.01) compared to the CT group. The eye area in the BG group was significantly increased (*p* < 0.05) compared to the SBM group. Also, there was a significant difference in the swim bladder area in the SBM group (*p* < 0.001) and BG group *(p* < 0.05) compared to the CT group. In the BG group, however, the effect was less pronounced, since swim bladder area was significantly increased (*p* < 0.001) compared to the SBM group (**Figure 1H**). We found a significant reduction in the head-trunk angle of the larvae from the SBM group (*p* < 0.05) compared with the CT group (**Figure 1I**). On the other hand, the head-trunk angle was increased in the BG group compared to the SBM group (*p* < 0.01).

**Figure 2:**
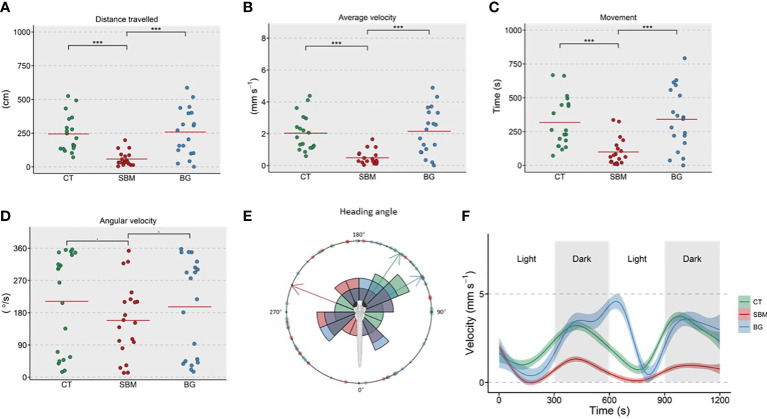
Changes in parameters linked to locomotor activity of zebrafish larvae. The measured parameters included **(A)** Distance travelled **(B)** Average velocity **(C)** Movement **(D)** Angular velocity and **(E)** Heading angle. Effect of alternating light-dark phases on the **(F)** velocity of zebrafish larvae. *** *p* < 0.001 and (.) *p* < 0.1. Larvae assessed at 15 dpf (n = 18-20 per group). Control - CT, soybean - SBM and β-glucan - BG.

The locomotor activity of the experimental larvae was evaluated by conducting a light-dark (LD) locomotion test. The total distance travelled (**Figure 2A**) by the SBM group was significantly reduced (*p* < 0.001) at 15 dpf. Average velocity (**Figure 2B**) and movement (**Figure 2C**) were also significantly (*p* < 0.001) reduced in the SBM group compared to both the CT and BG groups. The parameter angular velocity (**Figure 2D**) did not appear to be altered compared to the CT group (*p* < 0.1). However, the heading angle seemed to be slightly, but not significantly, altered in the SBM group (**Figure 2E**). The velocity vs. time plot (**Figure 2F**) of the light-dark phase experiment clearly indicated the loss in the dark-induced motion phenotype in the SBM group compared to the CT group. The BG group also showed a slightly altered activity after the first dark phase.

**Figure 3:**
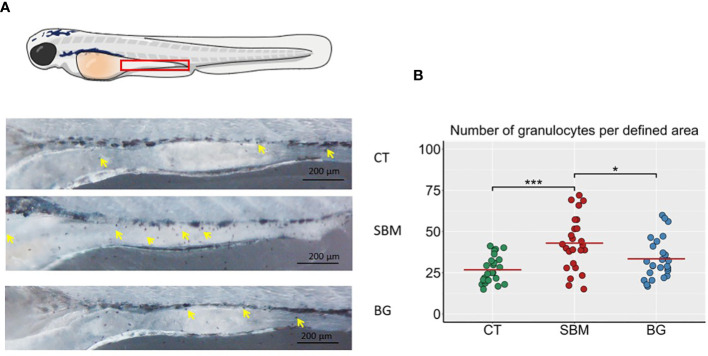
Localization of granulocytes in the intestine of zebrafish larvae. **(A)** Representative images of the intestine region of zebrafish larvae stained with Sudan Black to reveal the presence of granulocytes, yellow arrows indicate the granulocytes in the intestine region. **(B)** Quantification of Sudan-Black^+^ cells in the intestine region of zebrafish larvae. *** *p* < 0.001 and * *p* < 0.05. n = 24-25 per group. Control - CT, soybean - SBM and β-glucan – BG. Scale bar = 200 μm.

The larvae were stained with Sudan Black to examine the number of granulocytes in the intestine. The SBM diet-fed larvae had a greater number of granulocytes (*p* < 0.001) compared to the CT group. The BG group had lower number of granulocytes (*p* < 0.05) compared to the SBM group (**Figures 3A, B** **)**.

**Figure 4:**
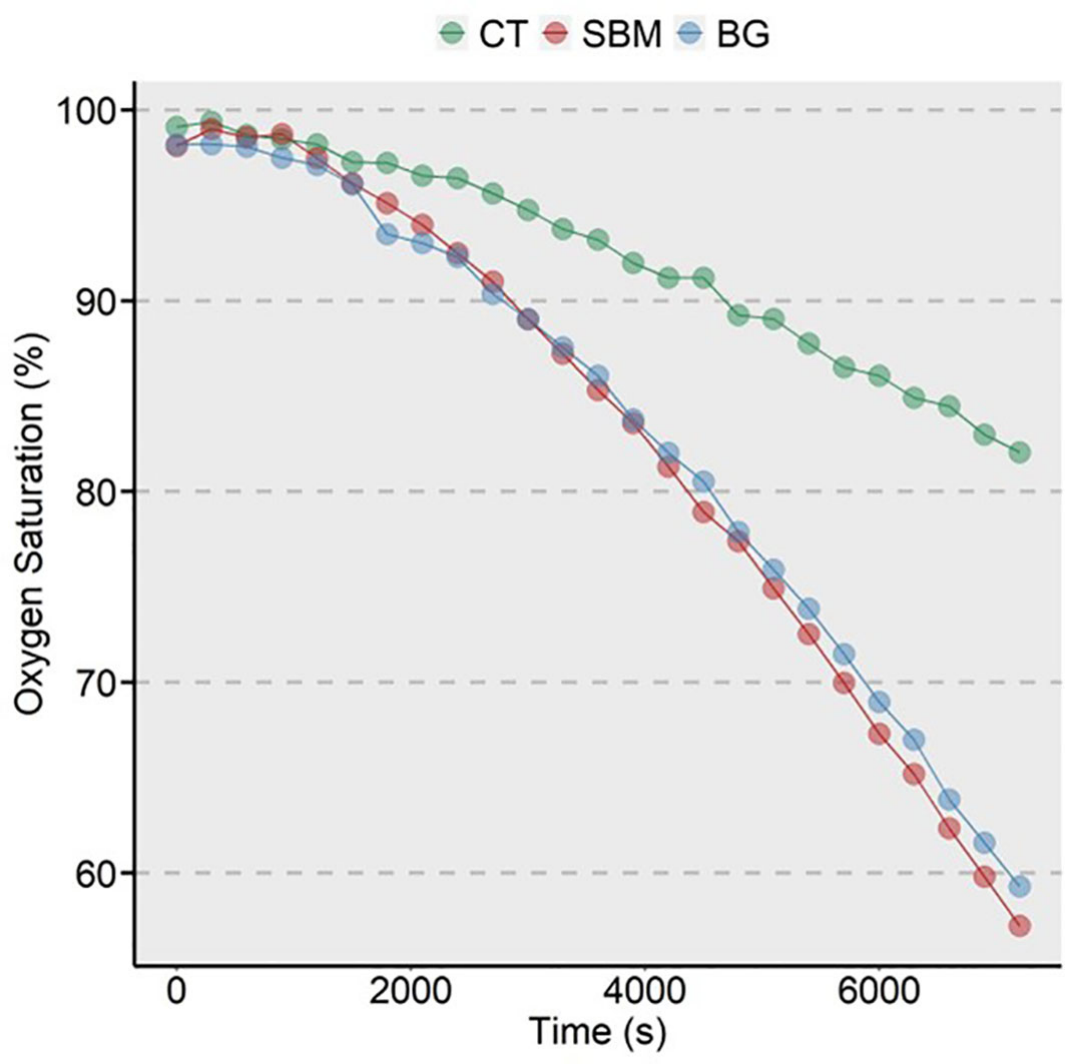
Oxygen consumption of zebrafish larvae from the 3 study groups. A generalized additive model using R package *mgcv* indicated significant differences (*p* < 0.05) in oxygen saturation. Larvae assessed at 15 dpf (n = 12 per group). Control - CT, soybean - SBM and β-glucan - BG.

We found an overall decrease in oxygen saturation with time in all the treatments. The oxygen saturation decreased at a significantly higher rate in the SBM and BG groups than in the CT group (**Figure 4**). The factors treatment and time were found to be significant (*p* < 0.05).

### Suppression of genes linked to visual perception and organ development

**Figure 5:**
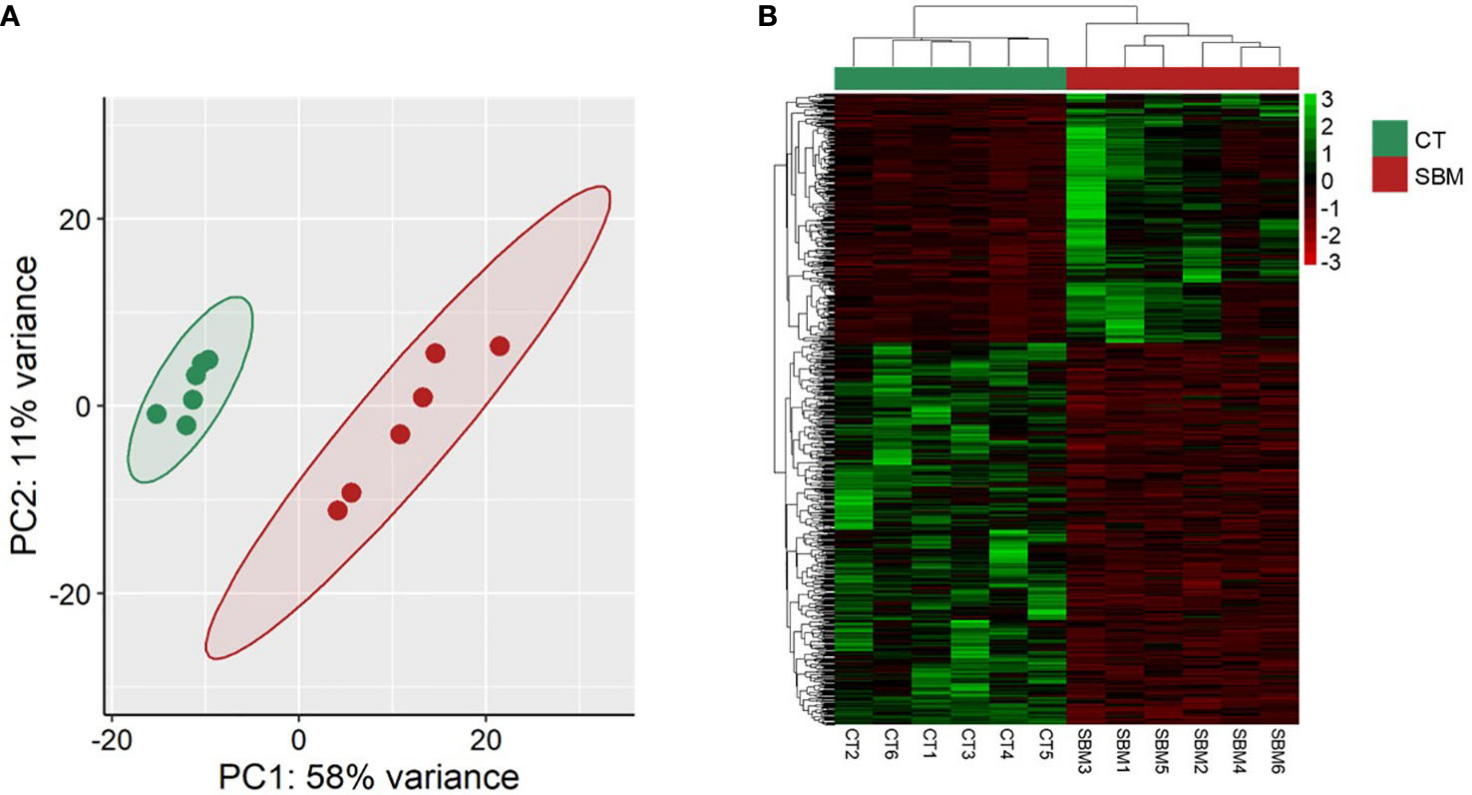
Transcriptome-based differences in zebrafish larvae from the soybean group compared to the control group. Principal component analyses **(A)** and heatmap **(B)** of the differentially expressed genes (DEGs) in the soybean (SBM) group compared to the control (CT) group. Transcripts with an adjusted *p*-value below 0.05 and |Log2 fold change| ≥ 1 were considered significantly differentially expressed. There are six biological replicates in each study group.

**Figure 6:**
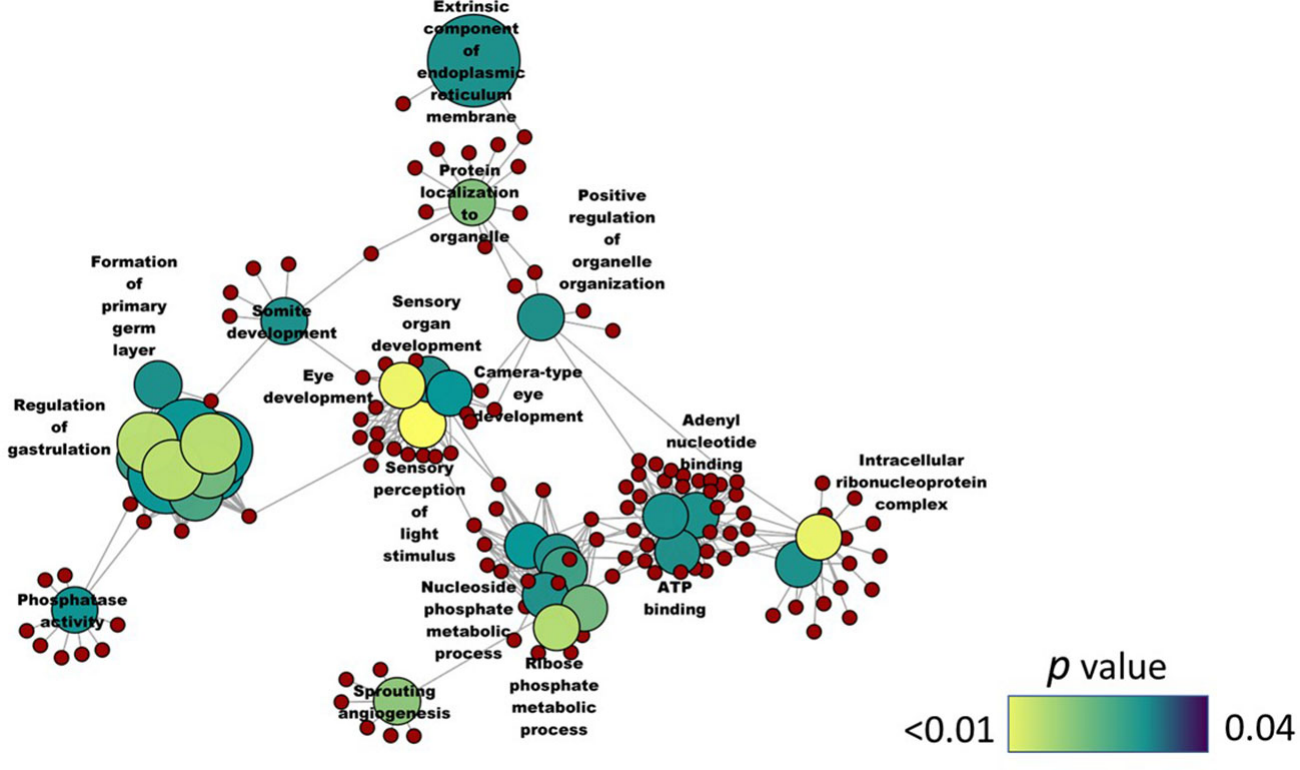
Network plot showing the link between the enriched GO terms. DEGs (downregulated; SBM vs CT) that were considered for the enrichment are indicated using red circles and only the non-redundant GO terms are shown in the cluster. The gradient color bar intensity varies with the *p* value and the sizes of the nodes of the GO terms increase with the associated fold change. There are six biological replicates in each study group. Control - CT, soybean - SBM.

**Figure 7:**
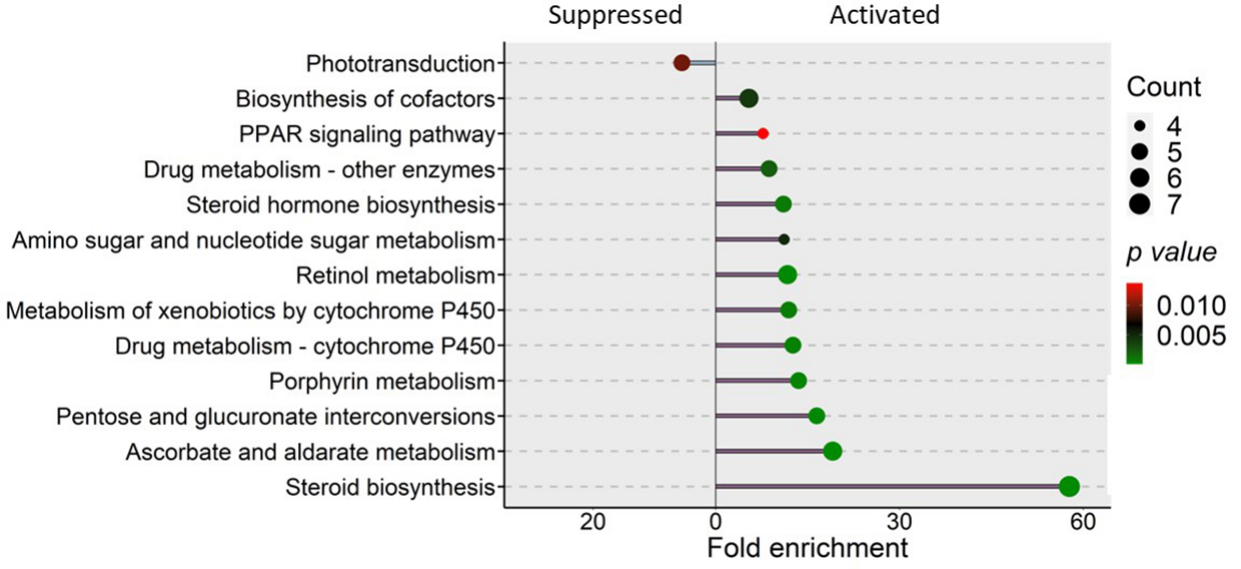
KEGG pathways that were enriched in the zebrafish larvae from the soybean group. Differentially expressed genes in the soybean (SBM) group compared to the control (CT) group were employed for the pathway enrichment analysis. The size of the circles is proportional to the gene count and gradient color bar intensity of circle correlates with the *p* value. There are six biological replicates in each study group.

**Figure 8:**
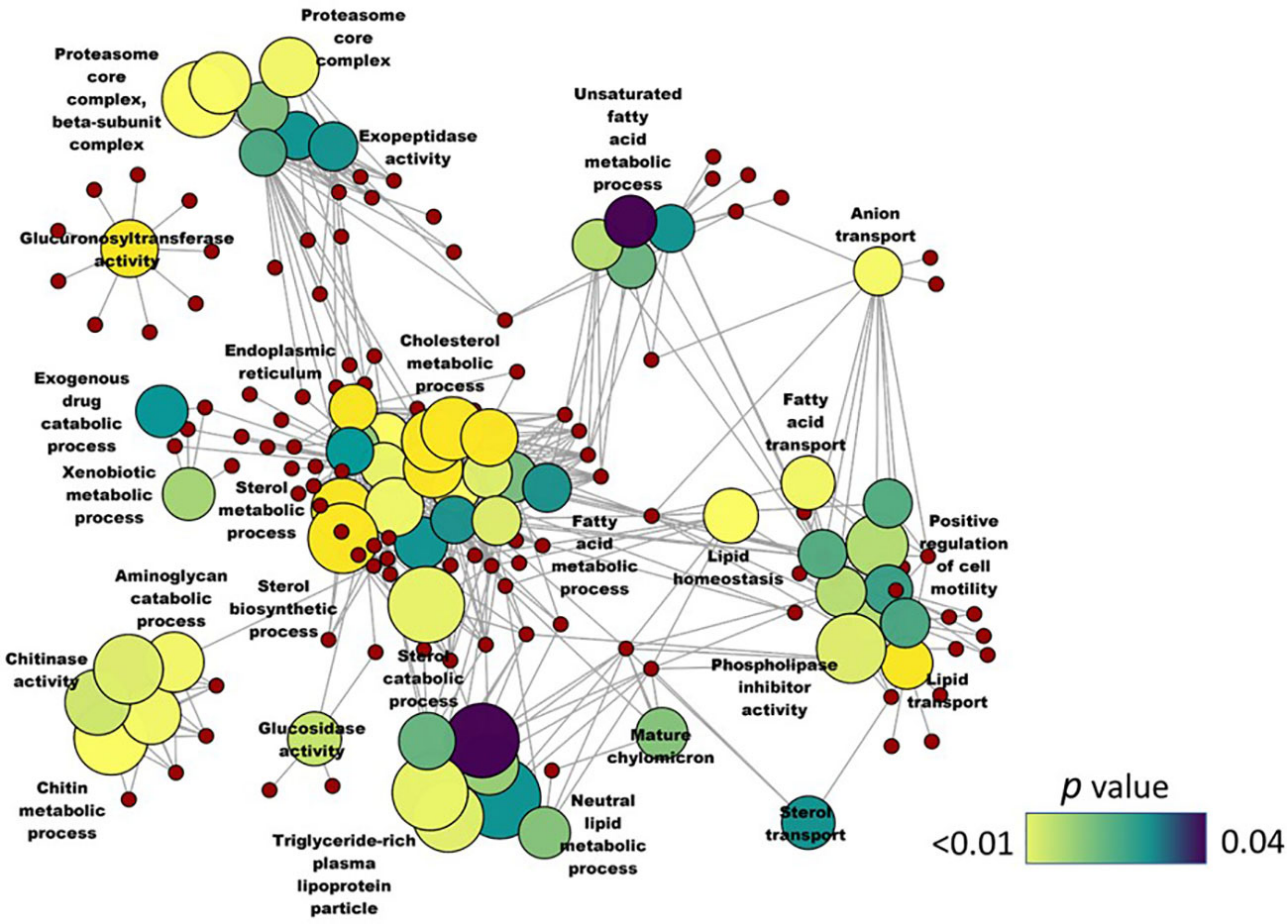
Network plot showing the link between enriched GO terms. DEGs (upregulated; SBM vs CT) that were considered for the enrichment are indicated using red circles and only the non-redundant GO terms are shown in the cluster. The gradient color bar intensity varies with the *p* value and the sizes of nodes of the GO terms increase with the associated fold change. There are six biological replicates in each study group. Control - CT, soybean - SBM.

To understand the underlying effects of the soybean feeding in zebrafish larvae, we compared the transcriptome of the SBM group with that of the CT group. The analysis revealed 707 differentially expressed genes (|Log_2_ fold-change| ≥ 1, adjusted *p*-value < 0.05) with 280 upregulated genes and 427 downregulated genes in the SBM group (**Supplementary Table 3**). The principal component analysis (PCA) plot shows the differential clustering of the SBM and CT groups along the first principal component (PC1), which explains 58% variability in the data (**Figure 5A**). Hierarchical clustering (**Figure 5B**) revealed a clear separation of differentially upregulated and downregulated genes in the SBM group compared to the CT group. The downregulated genes-associated significantly enriched GO terms in the SBM group were related to developmental processes like regulation of gastrulation, formation of primary germ layer, somite development and positive regulation of organelle organization (**Figure 6** **).** Furthermore, several GO terms related to sensory perception like sensory organ development, eye development, sensory perception of light stimulus and camera type eye development were also enriched based on the downregulated genes. KEGG enrichment analysis that considered the downregulated genes revealed the alteration of the phototransduction pathway in the SBM group compared to the CT group (**Figure 7**). On the other hand, upregulated genes-based KEGG pathway enrichment revealed the possible alteration of pathways like steroid biosynthesis, peroxisome proliferator activated lipid receptor (PPAR) signaling and metabolism of xenobiotics by cytochrome P450 (**Figure 7**). Similarly, upregulated genes-based GO enrichment analysis indicated the likely alteration of steroid biosynthesis process, sterol metabolic process, cholesterol metabolic process, fatty acid transport and lipid homeostasis **(** **Figure 8**). Furthermore, there was a significant enrichment of GO terms (based on the upregulated genes in the SBM group) related to protein degradation, namely proteasome core complex, exopeptidase activity and aminoglycan catabolic process **(** **Figure 8** **)**. Several immune, intestinal barrier and brain-related genes were altered in the SBM group compared to the CT group (**Supplementary Figures 3A-C**).

### β-glucan supplementation-caused distinct changes in zebrafish larvae

**Figure 9:**
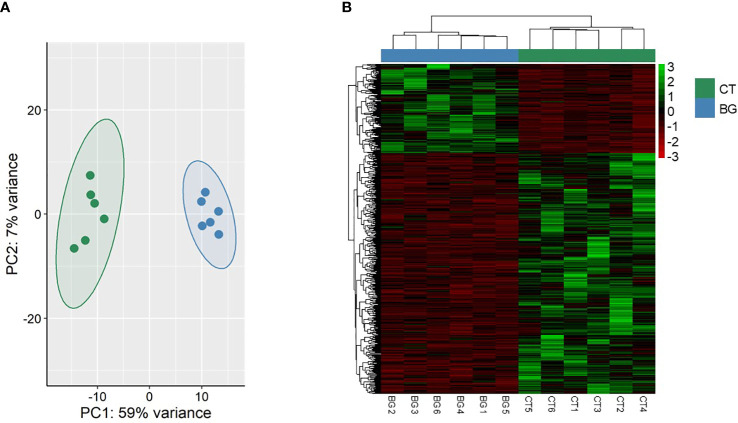
Transcriptome-based differences in the zebrafish larvae from the β-glucan group compared to the control group. Principal component analyses **(A)** and heatmap **(B)** of differentially expressed genes (DEGs) in the β-glucan (BG) group compared to the control (CT) group. Transcripts with an adjusted *p* below 0.05 and |Log2 fold change| ≥ 1 were considered as significantly differentially expressed. There are six biological replicates in each study group.

**Figure 10:**
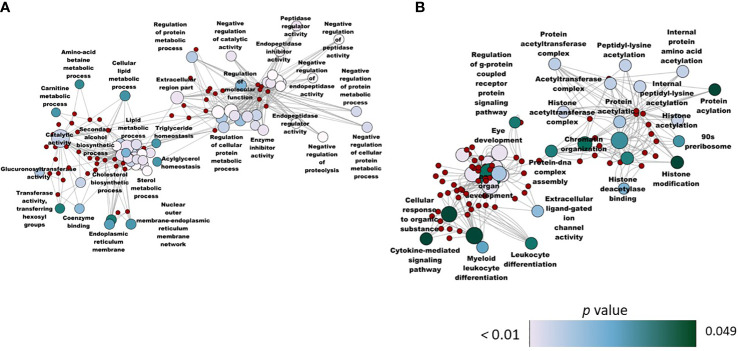
Network plot showing the link between the enriched GO terms. The enriched GO terms that were unique (from the BG and SBM vs CT comparisons) to the zebrafish larvae fed with the BG diet are shown in the figure, and 298 and 95 genes were significantly **(A)** upregulated and **(B)** downregulated, respectively, in BG vs CT comparison. Control - CT, soybean - SBM and β-glucan - BG.

Here we describe the DEGs from the BG vs CT comparison. As we did not detect any DEGs from the SBM vs BG comparison, unique (upregulated and downregulated) genes from the SBM vs CT and BG vs CT comparisons are given importance in this section. In addition, we selected the genes linked to the immune system to gather more evidence on the effect of β-glucan. A comparison of the transcriptome of the BG group with that of the CT group revealed 736 DEGs (|Log_2_ fold-change| ≥ 1, adjusted *p* value < 0.05) with 537 downregulated genes and 199 upregulated genes **(** **Supplementary Table 4**). The PCA plot shows differential clustering of the BG and CT groups along PC1, which explains 59% variability in the data (**Figure 9A**). Hierarchical clustering (**Figure 9B**) revealed a clear separation of differentially upregulated and downregulated genes in the BG group compared to the CT group. BG vs CT transcriptomic comparison revealed several common genes which were also present in the SBM vs CT comparison, possibly due to the presence of soybean meal in the BG diet too. We used a Venn diagram to display the common and unique DEGs from the SBM vs CT and BG vs CT comparisons (**Supplementary Figure 4** **)**; 343 common DEGs (239 downregulated and 104 upregulated) and 298 unique downregulated genes and 95 unique upregulated genes from the BG vs CT comparison. To reveal the efficacy of the β-glucan, we focused on the altered genes that are unique to the BG vs CT comparison. GO term enrichment by considering 95 unique upregulated genes included negative regulation of proteolysis, endopeptidase inhibitor activity and negative regulation of cellular protein metabolic process (**Figure 10A**). GO term enrichment based on the 298 unique downregulated genes in the BG group included cytokine mediated signaling pathway, leukocyte differentiation, histone acetyltransferase complex, histone acetylation, histone modification and regulation of G-protein coupled receptor protein signaling pathway (**Figure 10B**).

From the DEGs of the three comparisons, immune-, barrier- and brain-related genes were selected to understand the inflammation mitigation capacity of β-glucan. We observed that BG feeding restored the expression of several genes which were perturbed by SBM feeding (**Supplementary Figures 3A-C**). The expression of immune genes like *major histocompatibility complex class I UBA* (*mhc1uba*), *complement C3a* (*c3a.3*), *interleukin 2 receptor, gamma a* (*il2rga*), *macrophage stimulating 1 receptor a* (*mst1ra*) that was upregulated in the SBM group was downregulated in the BG group. The expressions of brain-related genes like *tachykinin receptor 1b* (*tacr1b*)*, bradykinin receptor b1* (*bdkr1*) in the BG group were similar to those in the CT group.

### A defective swim bladder persisted despite the cessation of soybean feeding

A switch to the control diet without soybean meal helped in restoring the phenotypic characteristics, locomotor activity and oxygen consumption of the SBM group. The locomotor behavioral analysis revealed that all the study groups performed similarly at 30 dpf (**Supplementary Figures 5A-D**), i.e., when the fish did not consume soybean meal-based diets. However, the head to trunk angle in the SBM group was significantly decreased (*p* < 0.05) compared to CT and BG groups. The swim bladder area in the SBM group was significantly smaller (*p* < 0.001) compared to the CT and BG groups. Notably, the swim bladder area in the BG group seemed to have returned to a level similar to the CT group (**Supplementary Figures 5E**, **6**). The oxygen consumption in all the three treatments at 30 dpf did not differ significantly (**Supplementary Figure 7**), even though we observed a steep decline in oxygen saturation in all the three treatments, indicating a higher consumption of oxygen at 30 dpf.

## Discussion

Soybean meal-based diet is effective in inducing intestinal inflammation in zebrafish. The attributes of inflammation in this model are increased intestinal permeability, immune cell recruitment and alteration in the microbiota profile (13–15). Behavioral changes accompanying diet-induced intestinal inflammation in animals have been reported previously (30, 31). In the present study, we have linked behavioral changes of zebrafish with alterations in the transcriptome to gain deeper insights into the impact of inflammatory and anti-inflammatory diets. Dietary soybean was found to reduce the locomotor activity, induce developmental defects and increase oxygen consumption of zebrafish larvae. Transcriptomic analysis indicated the soybean meal-induced suppression of the genes linked to visual perception, organ development, phototransduction pathway and activation of genes related to steroid biosynthesis pathway. On the contrary, β-glucan partly negated the behavioral and phenotypic alterations brought about by the soybean diet.

### Soybean-induced alteration in locomotor behavior

We performed a light-dark locomotion test to understand soybean meal-induced behavioral changes in zebrafish larvae. Alternating light and dark conditions prompts zebrafish to follow a specific pattern of movement; while a transition from light-dark increases locomotion, a dark-light transition decreases its movement (32). The CT and BG groups displayed normal responses to light, as indicated by the velocity-time analysis. On the other hand, the behavior of the SBM group was abnormal, probably due to a defect in sensory perception, as indicated by the potential shift in sensory organ development, sensory perception of light stimulus and the significantly affected phototransduction pathway. This pathway occurs in the retinal photoreceptors, namely rods and cone cells which are active at low and high light intensities, respectively (33). These cells convert light stimulus into electrical signals which are then perceived by the nervous system. Several genes related to light perception including *visual system homeobox 1* (*vsx1*), *recoverin a* (*rcvrna*) and *guanylate cyclase activator 1b* (*guca1b*) were downregulated in the SBM group. The proteins coded by these genes are critical for retinal functions like terminal differentiation of retinal cells and cone photo response recovery (34, 35). The transcriptomic data also revealed the potential alteration of several pathways such as retinol metabolism, steroid biosynthesis, PPAR signaling based on the upregulated genes in the SBM group. Alterations in these pathways have been linked with retinal impairments (36–38). In addition, SBM feeding reduced the eye area, indicating a possible impact on the overall eye development. Therefore, it can be speculated that the metabolic changes induced by the soybean-based diet might have affected retinal functions. The altered heading angle and angular velocity of the SBM group can also be linked to the impaired light perception. Furthermore, several locomotor behavioral parameters like distance travelled, velocity and movement were also decreased in the SBM group. Other feeding studies have also reported diet-induced changes in locomotion; while a high-fat, low-fiber diet reduced the motor activity of mice (30, 39) a probiotic diet (with *Lactobacillus rhamnosus* IMC 501) increased the movement of zebrafish (40). This indicates that locomotor behavior of animals can be altered by diet. Concerning the β-glucan diet, another study has indicated the efficacy of the compound in improving the cognitive ability in diet-induced colitis model of mice (9). The observations in the present study also likely indicate an improvement in locomotor behavior. Diet-induced behavioral changes can be due to microbiota alterations which in turn affect the gut-brain axis as observed in mice models (9). Although in the present study we did not investigate the microbiota changes, future studies should focus on the effect of diet-induced inflammation on the gut-brain axis.

### Soybean-induced developmental defects and oxygen demand

We did not detect any significant differences in growth parameters like standard length and snout to vent length in zebrafish larvae belonging to the 3 study groups. The short duration of the experiment, i.e., 10 days may not be sufficient to reveal any significant differences in growth, as observed in a study on red seabream, *Sparus aurata* larvae (41). We found a significant reduction in swim bladder area and head-trunk angle in the SBM group. The swim bladder is an important organ to maintain buoyancy, and larvae with uninflated swim bladders develop complications such as spinal deformities and lordosis (38). GO terms such as regulation of gastrulation, somite development, positive regulation of organelle organization and formation of primary germ layer likely indicate developmental defects of the larvae in the SBM group. As soybean meal-induced inflammation causes morphological changes in the intestine (42), and the swim bladder develops as an evagination of the digestive tract, there is a possibility that dietary soybean meal is interfering with swim bladder inflation. Furthermore, metabolic demands are higher in zebrafish larvae with uninflated swim bladders and these larvae use additional energy to maintain buoyancy (38, 39). The altered locomotor behavior and high oxygen consumption that we observed in the SBM group is likely due to uninflated swim bladder (43, 44). Furthermore, feeding CT diet to 15-30 dpf larvae of the 3 groups indicated that the SBM-caused adverse effects on behavior-related parameters and oxygen consumption rate can be abated by stopping the SBM diet. Although the swim bladder area was increased in SBM group at 30 dpf as compared to 15 dpf, it was still significantly smaller compared to the other treatment groups. It seems that developmental defects such as uninflated swim bladder and reduced head-trunk angle persist even after stopping the SBM diet. Addition of β-glucan speeds up the recovery or lowers the effect of SBM on development. This suggests that proper nutrition during the critical developmental window is essential to avoid long-lasting effects on an organism (45).

KEGG pathway analyses revealed steroid biosynthesis (most enriched pathway based on *p* value and gene count) and steroid hormone biosynthesis as the enriched pathways, based on the upregulated genes in the SBM group. We also found enrichment of several GO terms like cholesterol metabolic process, sterol biosynthesis process, and sterol metabolic process. This can be because plant-derived food has no cholesterol and soybean contains phytosterols which can reduce dietary cholesterol absorption (46). Such a scenario can lead to stimulation of cholesterol biosynthesis, as reported in a study on fish fed soybean meal (47). Cholesterol is the key precursor of steroid hormone biosynthesis and cholesterol biosynthesis is an oxygen-intensive process that requires 11 molecules of O_2_ per molecule of cholesterol (41). Furthermore, molecular oxygen is a prerequisite to most of the enzymatic reactions in the steroid biosynthesis pathway (45). Therefore, the higher consumption of oxygen could be attributed to the increased metabolic demands in the SBM and BG groups and steroid biosynthesis can be a causative factor.

### Plausible effects of dietary β-glucan on zebrafish larval transcriptome

Several immune, intestinal barrier and brain-related genes were altered in the SBM and BG groups compared to the CT group. In this section we describe the common and unique genes from the SBM and BG vs CT comparisons. It was only in the BG group that the differential expression of *jak1*, *zbtb11*, *jagn1a*, *lepr*, *il13ra2*, *ccl34b.1* caused an enrichment of the GO terms, leukocyte differentiation and cytokine mediated signaling. Janus Kinase (JAK1) plays an important role in inflammatory cytokine signaling and inhibition of JAK1-mediated inflammatory pathways are effective therapeutic targets to counter intestinal inflammation (48, 49). Furthermore, genes such as *leptin receptor* (*lepr*)*, jagunal homolog 1-A* (*jagn1a*) that are related to neutrophil development and migration (50, 51) and inflammation-associated cytokine like *il13ra2* (52) were differentially downregulated in the BG group. Granular cells (mainly neutrophils) are the first responders that migrate to an inflammatory site (53).The number of granulocytes in the BG group was lower compared to the SBM group, indicating the dietary β-glucan-mediated lowering of inflammation. In addition, the BG group was associated with GO terms related to negative regulation of proteolysis and endopeptidase inhibitor activity, as a result of the upregulated genes such as *serpin peptidase inhibitor, clade B, member 1, like 3* (*serpinb1l3*), *TIMP metallopeptidase inhibitor 4, tandem duplicate 2 (timp4.2)*. Serpin family B member is produced by macrophages and neutrophils to restrict the activity of serine proteases and inflammatory caspases to suppress inflammation (54). Also, tissue inhibitors of metalloproteinases (TIMPs) regulate diverse processes such as tissue remodeling, wound healing and inhibition of matrix metalloproteinases (46). Therefore, the upregulation of *serpinb1, serpinb1l3* and *timp4* in the β-glucan fed group might possibly help in controlling the tissue damage caused by dietary soybean. Moreover, the enriched GO term, G-protein-coupled receptor (GPCR) based on the downregulated genes, *ubiquitin-specific protease 20* (*usp20*) and *phosducin* (*pdca*) in the BG group is likely pointing to the efficacy of the glucan to counter inflammatory pathways. The protein coded by *usp20* is involved in the Tumor necrosis factor (TNFα)-induced activation of Nuclear factor kappa-light-chain-enhancer of activated B cells protein (NF-κB) pathway through the stabilization of p62 protein (55). In addition, β-glucan is also a potent epigenetic modulator (50, 51). Several GO terms related to epigenetic modifications such as histone acetyltransferase complex, histone modifications, chromatin organization were enriched (based on downregulated genes) in the BG group. Histone acetyltransferases (HATs) transfer acetyl groups from donor-acetyl coenzyme A to lysine residues of the histone proteins to sustain an active transcription. HATs also act as a cofactor for NF-κB activation by acetylating its various promotor proteins (56) because a HAT knockout study reported reduced DSS-induced colitis in mice (57). The expression of immune genes like *mhc1uba*, *c3a.3*, *il2rga*, *mst1ra* were upregulated in the SBM group, but the CT and BG groups had similar mRNA levels. Two brain-related genes, *tachykinin receptor 1b* (*tacr1b)* and *bradykinin receptor b1* (*bdkr1*) that were upregulated by the SBM diet (58, 59) were found to be downregulated by the BG diet. Therefore, we speculate that β-glucan can also reduce intestinal inflammation induced by the soybean diet, plausibly by altering the expression of GPCRs, cytokine signaling and inducing epigenetic modifications.

## Conclusion

Our study shows that dietary soybean meal reduces larval locomotor behavior, increases oxygen consumption, and induces developmental defects in zebrafish larvae. Transcriptomic analysis indicated soybean meal-induced suppression of genes related to the phototransduction pathway, organ development and activation of genes linked to the steroid biosynthesis pathway. Dietary β-glucan can likely alleviate the behavioral defects induced by the inflammatory diet and negate the aforementioned alterations in gene expression. Importantly, when zebrafish larvae receive an inflammation-sustaining dietary component, the developmental defects persist even after the withdrawal of the inflammatory diet. It would be interesting to explore if the gut microbiota has a role in the observed alterations. Hence, future studies should focus on the effect of dietary soybean-induced inflammation on the gut-brain axis.

## Data availability statement

The datasets presented in this study can be found in online repositories. The name of the repository/repositories and accession number(s) are: NCBI, PRJNA867519.

## Ethics statement

The animal study was reviewed and approved by the Norwegian Animal Research Authority, FDU (Forsøksdyrutvalget ID-22992).

## Author contributions

SR, VK, SB and JD designed the study. JD prepared feeds, and SR and AG performed the feeding experiment. AG, SR and SV did the sampling. SR and SV performed the behavioral study and its data analysis. AG and SR performed the bioinformatic analysis. SR and VK wrote the manuscript. All authors reviewed the article and approved the submitted version.

## Funding

SR, AG and SV were supported by Netaji Subhas-ICAR International Fellowships (NS-ICAR IFs) from the Indian Council of Agricultural Research, India.

## Acknowledgments

We are highly grateful to our colleague Bisa Saraswathy for her guidance and support during the manuscript preparation. We want to thank Kemin Industries Inc, USA, for providing us the commercial product of algal β-glucan, Aleta^™^.

## Conflict of interest

Author JD was employed by company SPAROS Lda.

The remaining authors declare that the research was conducted in the absence of any commercial or financial relationships that could be construed as a potential conflict of interest.

## Publisher’s note

All claims expressed in this article are solely those of the authors and do not necessarily represent those of their affiliated organizations, or those of the publisher, the editors and the reviewers. Any product that may be evaluated in this article, or claim that may be made by its manufacturer, is not guaranteed or endorsed by the publisher.

## Supplementary material

Supplementary Figure 1 Experimental design of the study.

Supplementary Figure 2 Microscopic images of the zebrafish larve at 15 dpf. Scale bar = 500 μm.

Supplementary Figure 3 Heatmaps of mean normalized counts of selected differentially expressed genes associated with the 3 study groups **(A)** Immune **(B)** Barrier and **(C)** Brain-related differentially expressed genes (DEGs) were selected from different treatment groups.

Supplementary Figure 4 Venn diagram to display the common and unique differentially expressed genes from the soybean vs control, and β-glucan group vs control comparisons.

Supplementary Figure 5 Changes in parameters linked to locomotor activity and morphology of zebrafish larvae. The measured parameters included **(A)** Distance travelled **(B)** Average velocity **(C)** Movement. Effect of alternating light-dark phases on the **(D)** Velocity of zebrafish larvae **(E)** Head to trunk angle **(F)** Swim bladder area. Larvae assessed at 30 dpf (n = 18-20 per group).

Supplementary Figure 6 Microscopic images of the zebrafish larvae at 30 dpf. Scale bar = 500 μm.

Supplementary Figure 7 Oxygen consumption of zebrafish larvae from the 3 study groups. Larvae assessed at 30 dpf (n = 12 per group). Control - CT, soybean - SBM and β-glucan - BG.
