# Supplementary figures and images for: Developmental defects and behavioral changes in a diet-induced inflammation model of zebrafish
Source: Front Immunol. 2022 Oct 26;13:1018768. doi: 10.3389/fimmu.2022.1018768 (PMC9643868; doi:10.3389/fimmu.2022.1018768)

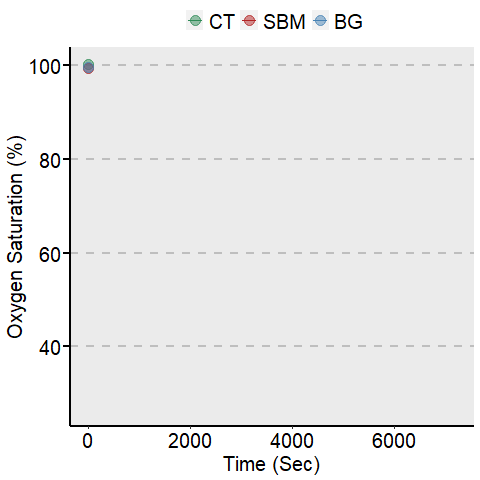

Supplement: Supplementary file 1 [file DataSheet_1.zip › Supplemetary Figures ZF_06_21_revised/Supplemenatry Figure 7.gif]

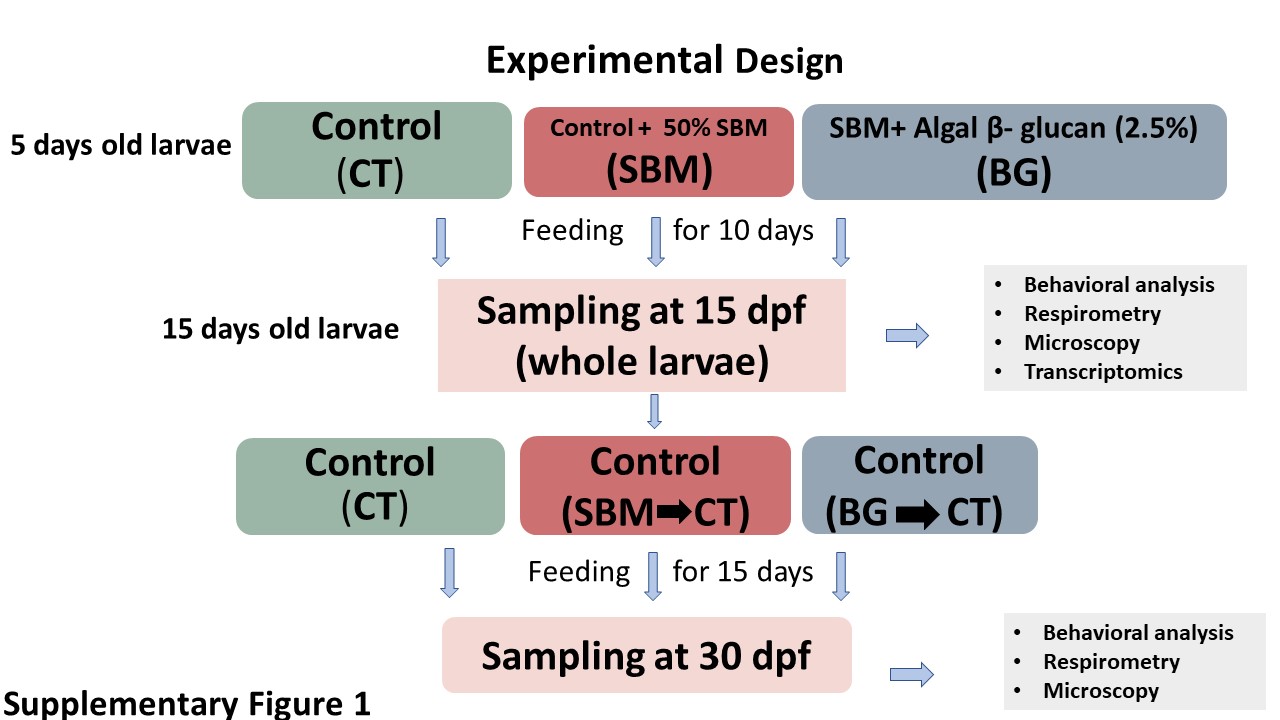

Supplement: Supplementary file 1 [file DataSheet_1.zip › Supplemetary Figures ZF_06_21_revised/Supplementary Figure 1.JPG]

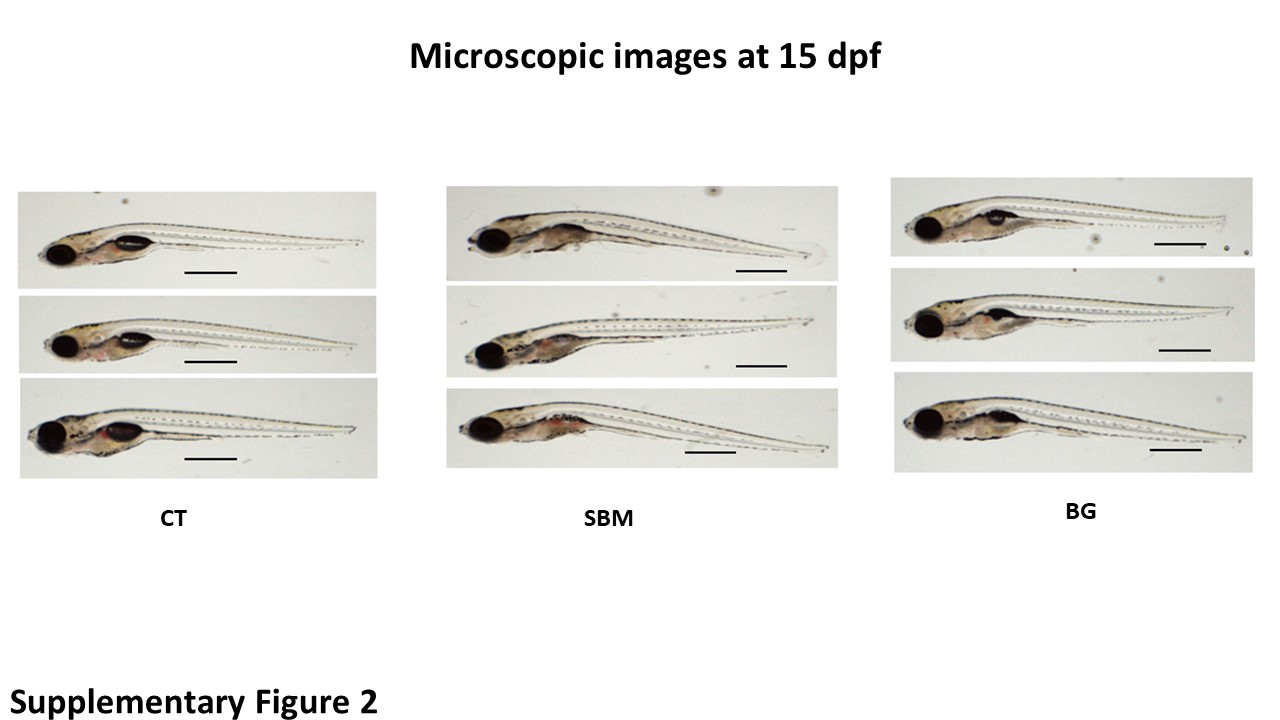

Supplement: Supplementary file 1 [file DataSheet_1.zip › Supplemetary Figures ZF_06_21_revised/Supplementary Figure 2.JPG]

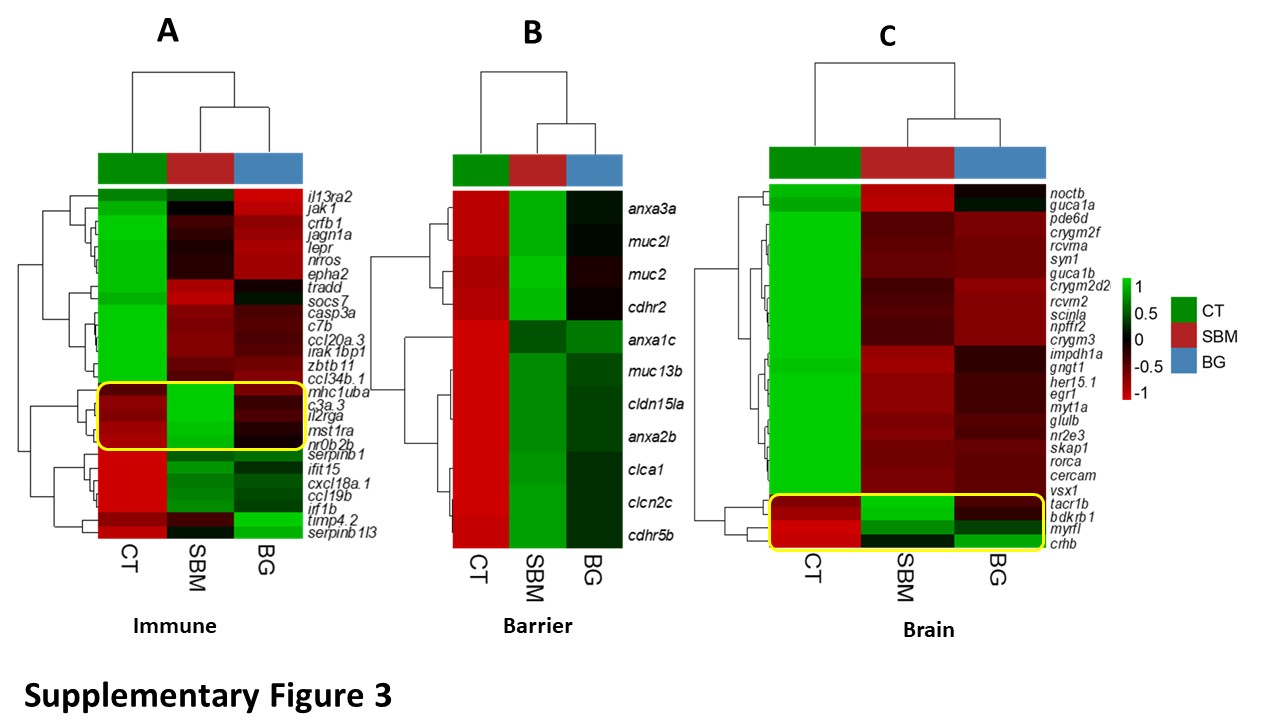

Supplement: Supplementary file 1 [file DataSheet_1.zip › Supplemetary Figures ZF_06_21_revised/Supplementary Figure 3.JPG]

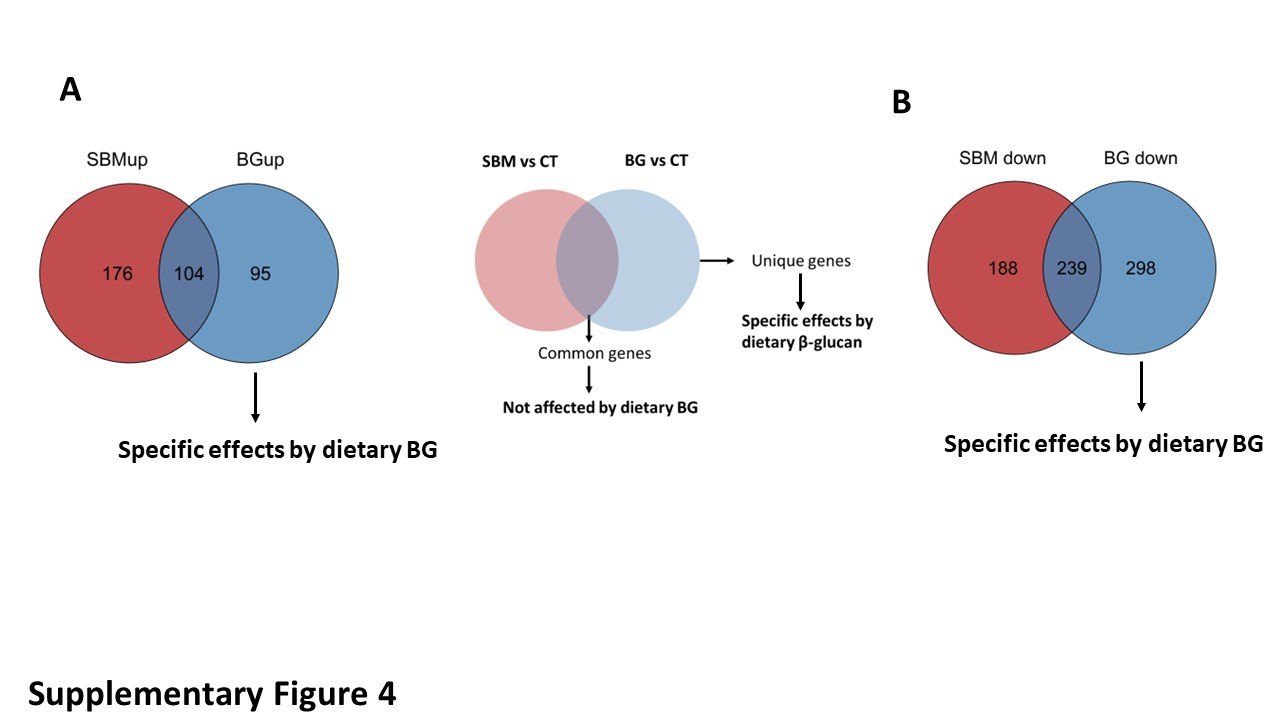

Supplement: Supplementary file 1 [file DataSheet_1.zip › Supplemetary Figures ZF_06_21_revised/Supplementary Figure 4.JPG]

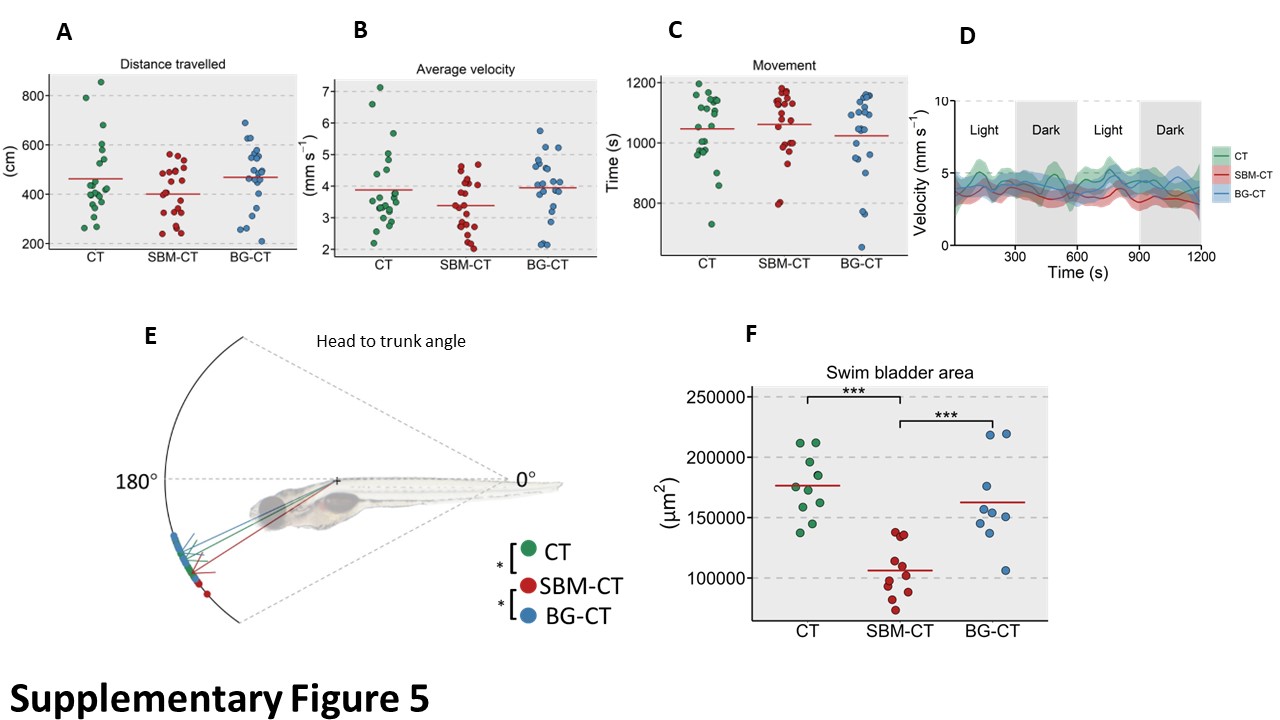

Supplement: Supplementary file 1 [file DataSheet_1.zip › Supplemetary Figures ZF_06_21_revised/Supplementary Figure 5.jpg]

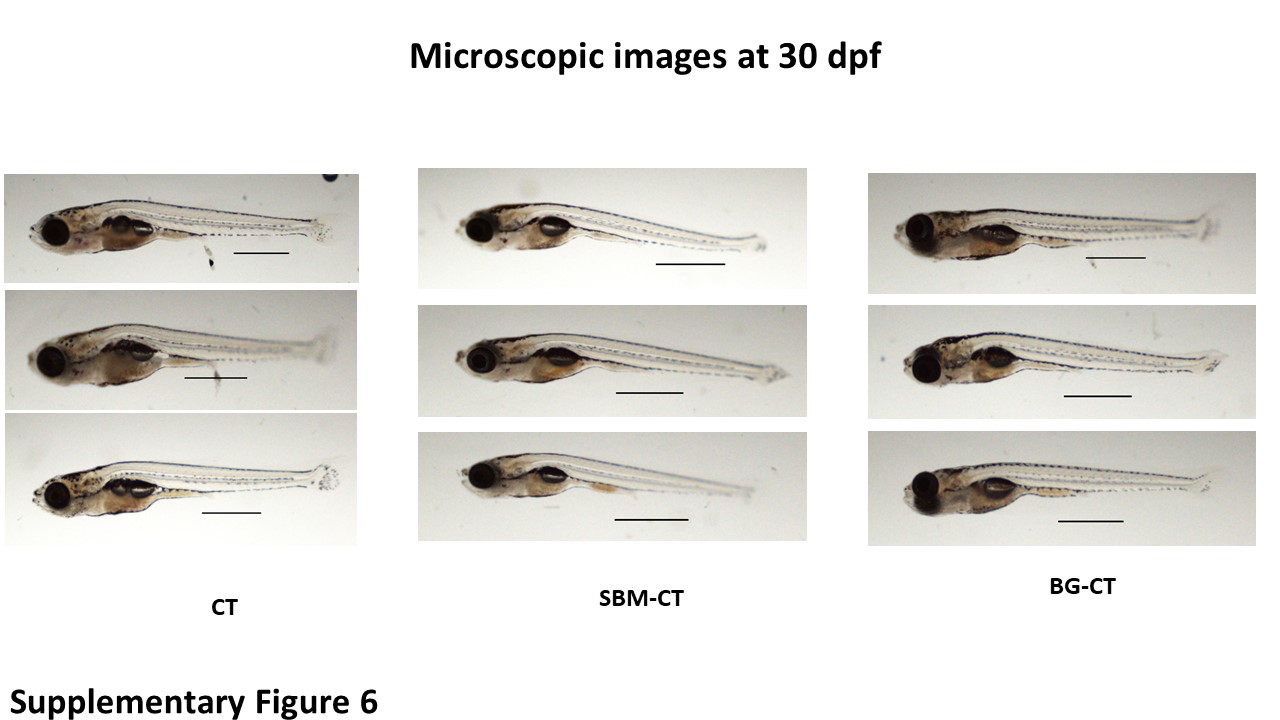

Supplement: Supplementary file 1 [file DataSheet_1.zip › Supplemetary Figures ZF_06_21_revised/Supplementary Figure 6.JPG]
